# Cell-Penetrating Peptide-Based Delivery of Macromolecular Drugs: Development, Strategies, and Progress

**DOI:** 10.3390/biomedicines11071971

**Published:** 2023-07-12

**Authors:** Zhe Sun, Jinhai Huang, Zvi Fishelson, Chenhui Wang, Sihe Zhang

**Affiliations:** 1 School of Life Sciences, Tianjin University, Tianjin 300072, China; sz2019226008@tju.edu.cn (Z.S.); jinhaih@tju.edu.cn (J.H.); 2 Department of Cell and Developmental Biology, Faculty of Medicine, Tel Aviv University, Tel Aviv 69978, Israel; lifish@tauex.tau.ac.il; 3 Department of Cell Biology, School of Medicine, Nankai University, Tianjin 300071, China; 15737371232@163.com

**Keywords:** cell-penetrating peptide, macromolecular drug delivery, cellular uptake mechanism, biological barrier, optimized strategy

## Abstract

Cell-penetrating peptides (CPPs), developed for more than 30 years, are still being extensively studied due to their excellent delivery performance. Compared with other delivery vehicles, CPPs hold promise for delivering different types of drugs. Here, we review the development process of CPPs and summarize the composition and classification of the CPP-based delivery systems, cellular uptake mechanisms, influencing factors, and biological barriers. We also summarize the optimization routes of CPP-based macromolecular drug delivery from stability and targeting perspectives. Strategies for enhanced endosomal escape, which prolong its half-life in blood, improved targeting efficiency and stimuli-responsive design are comprehensively summarized for CPP-based macromolecule delivery. Finally, after concluding the clinical trials of CPP-based drug delivery systems, we extracted the necessary conditions for a successful CPP-based delivery system. This review provides the latest framework for the CPP-based delivery of macromolecular drugs and summarizes the optimized strategies to improve delivery efficiency.

## 1. Introduction

**Figure 1:**
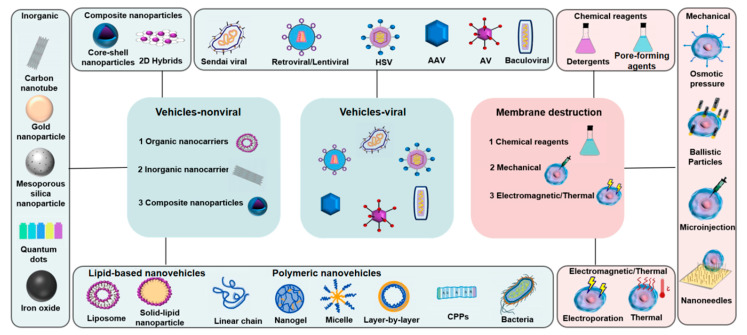
Delivery systems for macromolecular drugs.

How macromolecular drugs can be efficiently delivered to target cells remains a big challenge. There are two ways to place macromolecular drugs into target cells: one is to directly introduce the macromolecular drugs into cells based on membrane disruption; the other is to deliver them by using delivery vehicles [1], which are mainly divided into either viral vehicles or non-viral vehicles. Various macromolecular drug delivery systems are shown in Figure 1.

Delivery, based on membrane disruption, can quickly and directly deliver almost all macromolecular drugs that can be dispersed in a solution; however, it must also avoid cell disturbance or death caused by membrane destruction [1]. The delivery methods based on membrane disruption are mainly classified into three approaches: (1) electromagnetic/thermal; (2) mechanical; and (3) chemical. These approaches usually include electroporation [2], microinjection [3], osmotic pressure [4], nanoneedles [1], pore-forming agents [5], and more (Figure 1). The principle underlying these approaches is to briefly destroy the cell membrane so that macromolecular drugs can enter the cell, after which the cell membrane is repaired and can restore cell homeostasis. However, such an approach has strict requirements regarding the degree and time of membrane disruption, and it requires chemically modifying the macromolecular drugs to prevent their degradation [6]. Currently, the selection of a suitable membrane disruption method and its precise implementation in a high-throughput way remains challenging [1].

Viral delivery vehicles can overcome cellular barriers and can effectively deliver macromolecular drugs into cells. The most commonly used types of viral delivery vehicles are the retrovirus (RV), lentivirus (LV) [7], adenovirus (AV), and adeno-associated virus (AAV) [8]. LVs are a subtype of RVs and can be stably integrated into the host genome of mammalian cells [9]. Compared with the RV and AV, the LV and AAV are the most used vehicles in clinical trials because of their lower immunogenicity. However, the LV is prone to bring about higher off-target effects [10], and the drug-loading capacity of the AAV is also limited [11]. Aside from these vehicles, there are also herpes simplex virus (HSV) vehicles, with a larger packaging capacity [9], and the Epstein-Barr virus (EBV); EBV is subordinate to HSV, but it is also used as a delivery vehicle for CRISPR/Cas [12,13]. In addition, there are Sendai virus vehicles, which are used as a negative-strand RNA virus vehicle system [14], as well as the baculovirus, which is used as a gene expression vehicle [15] (Figure 1). Although viral vehicles have high delivery efficiency for macromolecular drugs, their limited drug-loading capacity, high production costs, uncontrollable viral release, limited viral tropism, and safety issues regarding viral gene integration into the host genome have still not been completely resolved [16].

Non-viral delivery vehicles have been widely explored in recent years because of their lower immunogenicity, unrestricted drug-loading, high flexibility, simple synthesis, low cost, and easy storage [17,18,19,20]. Non-viral delivery vehicles can be divided into three categories: (1) organic nanovehicles; (2) inorganic nanovehicles; and (3) composite nanoparticles [21]. Of these vehicles, organic nanovehicles include cell-penetrating peptides (CPPs), bacteria-derived or lipid-based nanovehicles, and polymeric nanovehicles (Figure 1). Since low delivery efficiency is a problem for most non-viral delivery vehicles [22], CPPs that can traverse the biological barrier (cell membranes) with high efficiency have been extensively explored. CPPs are not only simple to synthesize but are diverse in their types and are widely used [23]. They can also be combined with other delivery vehicles to further improve the delivery efficiency of macromolecular drugs [24,25,26]. Compared with the usual way of membrane disruption, the use of CPPs causes less damage to the cell membrane and is more effective and safe [27]. Therefore, CPPs are very useful tools for macromolecular drug delivery.

## 2. The History of CPP Development

CPPs usually comprise 5–30 amino acids with good biological safety and can efficiently carry macromolecular drugs through the cell membrane. The history of CPPs can be traced back to 1988. The first CPP was discovered when researching potential targets for the treatment of AIDS, namely, the Trans-Activator of Transcription (Tat) protein of human immunodeficiency virus type 1 (HIV-1) [28,29]. Then, in 1991, the homologous protein Penetratin/Antp from the antennae of Drosophila melanogaster was discovered [30]. The amphipathic penetrating peptide MPG [31], truncated HIV-Tat [32], and VP22, derived from HSV structural protein, were discovered in 1997 [33]. Then, the first chimeric peptide Transportan (TP) was reported in 1998 [34]. Subsequently, Oligo R [35], Pep-1 [36], and SS-31 [37] were discovered one after the other.

**Figure 2:**
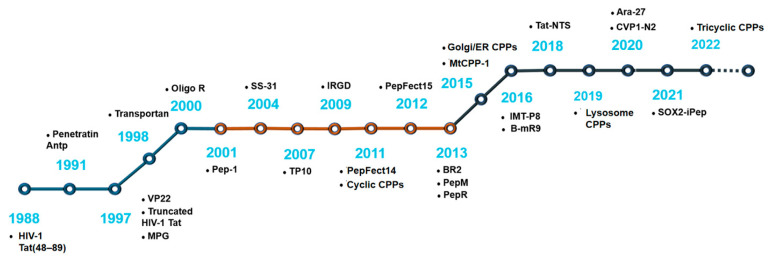
Milestones for the discovery and development of CPPs.

Transportan is a combination of the peptide mastoparan, isolated from wasp venom, and the N-terminal fragment of the human neuropeptide galanin. Transportan 10 (TP10), a chimeric peptide derived from mastoparan, comprises a 14-residue peptide from wasp (*Vespula lewisii*) venom linked to a 6-residue sequence from the neuropeptide galanin through an extra lysine residue, was developed in 2007 [38]. The tumor-targeting penetrating peptide iRGD was reported in 2009 [39]. In 2011, PepFect14 was reported to have improved the stability in serum [40]; PepFect15 was reported a year later [41]. Further research in 2013 found that the cancer-specific penetration peptide, BR2, can effectively deliver a single-chain antibody (scFv) [42]. Thereafter, PepM and PepR from the dengue virus capsid protein were discovered [43]. In recent years, the mitochondrial-penetrating peptide MtCPP-1 [44]; skin-permeable IMT-P8 [45]; bioreducible B-mR9 [46]; Tat-NTS, which can inhibit the transcriptional activity of *p53* [47]; VP1, derived from the VP1 protein of chicken anemia virus [48]; Ara-27, from Arabidopsis thaliana [49]; and peptide SOX2-iPep have been reported as successful [50]. Notably, various types of cyclic CPPs and intracellular organelle targeting CPPs have been newly developed [51,52,53]. Milestones in the development process of CPPs are depicted in Figure 2.

## 3. Classification of the CPP-Based Macromolecular Drug Delivery System

**Figure 3:**
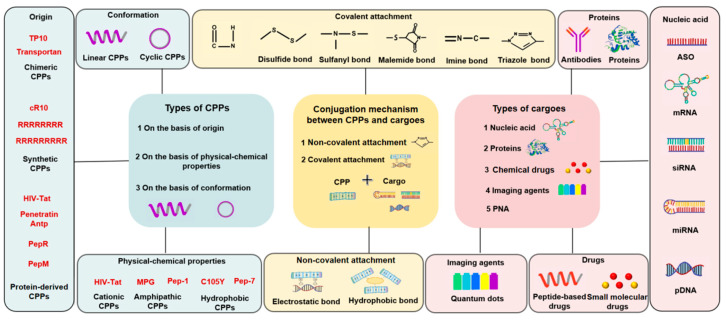
The components and classification of the CPP-based macromolecular drug delivery.

The components of the CPP-based delivery system include CPPs and delivered drug cargo. There are many types of CPPs and drug cargoes. CPPsite 2.0, a CPP database, currently holds around 1850 peptide entries and maintains more detailed information about different types of CPPs and delivered cargoes. The CPPsite 2.0 website can also be used to predict the tertiary structures of CPPs [54,55]. Below, we summarize the type, classification, and connection between the CPPs and the delivered drug cargoes (Figure 3).

### 3.1. Classification of CPPs

Generally, CPPs can be divided into three categories by their source, physical and chemical properties, and composition. According to the literature, CPPs can be divided into synthetic CPPs, chimeric CPPs, and protein-derived CPPs [56]. According to their physical and chemical properties, CPPs can be divided into cationic CPPs, amphipathic CPPs, and hydrophobic CPPs [57]. In addition, according to their composition, CPPs can be divided into cyclic CPPs and linear CPPs. Although they only occupy a small proportion (about 5%) compared with linear CPPs, cyclic CPPs reduce the degree of conformation freedom due to the peptide cyclization, which greatly improves their binding specificity with targeted molecules [58]. This stable structure of cyclic CPPs has greater resistance to proteolytic degradation [59]. Cyclic CPPs also perform better in terms of oral bioavailability [60]. In addition, cyclic CPPs can more efficiently interfere with protein–protein interactions (PPIs) than can traditional small-molecule drugs (MW ≤ 500) [61].

### 3.2. Classification of Cargos

Generally, macromolecular drug cargoes can be divided into nucleic acids, proteins, polymer chemicals or imaging agents, PNAs (peptide nucleic acids), and more (Figure 3). Small-molecule drugs include peptides and different types of chemicals. Macromolecular nucleic acid drugs mainly include antisense oligonucleotides (ASOs), mRNA, siRNA, miRNA, and pDNA. Macromolecular protein drugs mainly include different types of proteins and antibodies with functional activity. Protein-based therapy has a faster effect, better functional strength, and durable controllability; its lack of genetic toxicity has been extensively highlighted [62]. Moreover, the use of CPPs can promote the delivery of protein cargoes into cells non-invasively [63]. Because the delivery only by single CPPs has shortcomings of non-specificity, easy hydrolysis, and a short half-life [35,64], combining CPPs with other vehicles increases the delivery efficiency for macromolecular drugs [63]. In addition, accumulating research indicates that CPP-modified vehicles can be used to deliver other types of macromolecular-complexed drugs, including nanoparticles, liposomes, micelles, and cellulose-based superabsorbent hydrogels [65,66,67].

### 3.3. CPP and Cargo Connection Types

The CPP and cargo connection types can be divided into those with covalent bonds and those with non-covalent connections. The non-covalent connection methods mainly include hydrophobic interactions and electrostatic interactions [68]. The electrostatic interaction connection method has poor stability, which is highly dependent on the ionic strength and pH [69]. There are many types of covalent bond connection methods; these bonds include peptide bonds [70], disulfide bonds [71,72], sulfanyl bonds [35], maleimide bonds [73], imine bonds [73], and triazole bonds [74]. Covalent linkages of CPPs to cargoes provide high stability and precise control over site selectivity [21]; however, sometimes, the covalent connection method is not conducive to the release of cargo. Notably, ConjuPepDB, a database of peptide-drug conjugates, has been recently released. It covers more than 1600 peptide-conjugated drug molecules, along with basic information about their biomedical application and the type of chemical conjugation employed [75].

## 4. Cellular Uptake Mechanisms, Influencing Factors, and Biological Barriers

**Figure 4:**
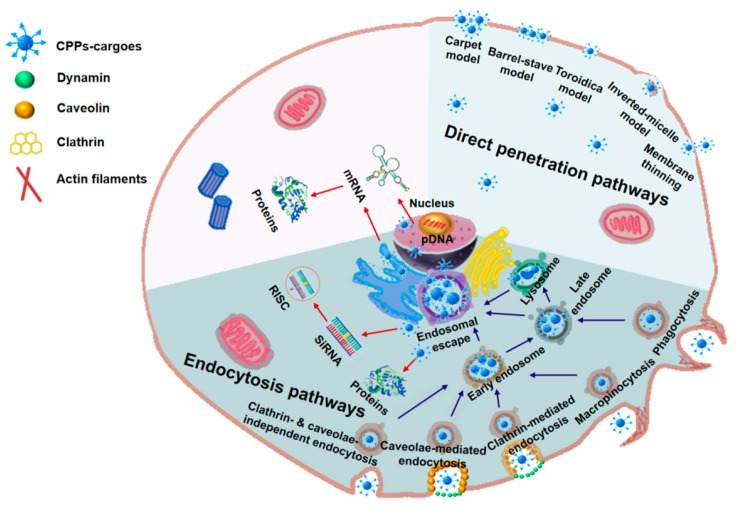
Cellular uptake mechanisms of CPP-based macromolecular drug delivery. The mechanisms mainly include two categories: direct penetration pathways and endocytotic pathways. Direct penetration pathways are usually energy-independent, whereas endocytotic pathways are energy-dependent. In order for the CPP-cargos to function after entering the target cells, proteins and RNA endosomes must escape into the cytoplasm, whereas pDNA must enter the nucleus.

Cellular uptake plays a key role in the efficient delivery of macromolecular drugs. CPPs can interact with the plasma membrane phospholipids, proteoglycans, and protein receptors on the cell surface that are taken up into cells [61]. The cellular uptake mechanisms of CPP-based macromolecular drug delivery mainly include two categories: direct penetration and endocytic routes. The penetrating peptide and the smaller penetrating peptide–cargo complex penetrate directly into the cell. The methods of direct penetration mainly include the carpet model [1], barrel-stave model [76], toroidal pore model [77], inverted micelle model [78,79], and membrane thinning [80]. Different macromolecular drugs have different endocytic pathways. The endocytic pathways of CPP-based macromolecular drug delivery mainly include five types: clathrin-mediated endocytosis (CME), caveolin-mediated endocytosis, autophagy pathway, macropinocytosis pathway, and caveolin- and clathrin-independent mode of endocytosis (Figure 4). These energy-dependent endocytic pathways transport most of the uptaken macromolecular drug cargoes to the early endosome, the late endosome, and finally to the lysosome. For the CPP cargoes to function after their uptake into cells, the delivered proteins and RNAs inside the endosomes must escape into the cytoplasm, whereas the delivered pDNA needs to enter the nucleus in order to function.

Different pathways of cellular uptake have distinct characteristics. It was reported that some viruses use caveolin-mediated endocytic pathways to avoid lysosome degradation [81,82,83]. CME is very important because it is the main way for cells to obtain nutrients; for example, by promoting the absorption of cholesterol and iron. However, the average diameter of clathrin-coated vesicles is about 100 nm, which limits the size of the cargo transported via this route [84]. Some studies also indicate that the macropinocytosis pathway can enhance the cellular uptake of macromolecular cargoes; this is conducive to the delivery of therapeutic genes [85]. The spread of the SARS-CoV-2 virus highlights the importance of research on the cellular uptake mechanism because a better understanding of the cellular uptake mechanism will help develop antiviral therapies [86]. However, research on the cellular uptake routes is still in the preliminary stage, and the reported uptake pathways of many macromolecular particles remain controversial.

Such complicated cellular uptake routes are also affected by many factors: the nature of the delivery system (size, shape, charge, and surface rigidity), the cell types, and the experimental factors (conditions such as the drug concentration, action time, and treatment temperature) [87]. Generally, small nanoparticles are easier for cellular uptake, and nanoparticles above 200 nm tend to accumulate in the spleen and liver [88,89]. However, if the size of the nanoparticles is less than 10 nm, they will be rapidly cleared by the kidneys and will not easily accumulate at the tumor site through the enhanced permeability and retention (EPR) effect [90,91,92]. In addition, positively charged nanomaterials exhibit higher cellular uptake than neutral or negatively charged nanomaterials. The harder the nanoparticle, the easier it is for it to be taken up by the cells [83]. Moreover, when the hydrodynamic size is the same, nanorods can penetrate better than nanospheres [93].

**Figure 5:**
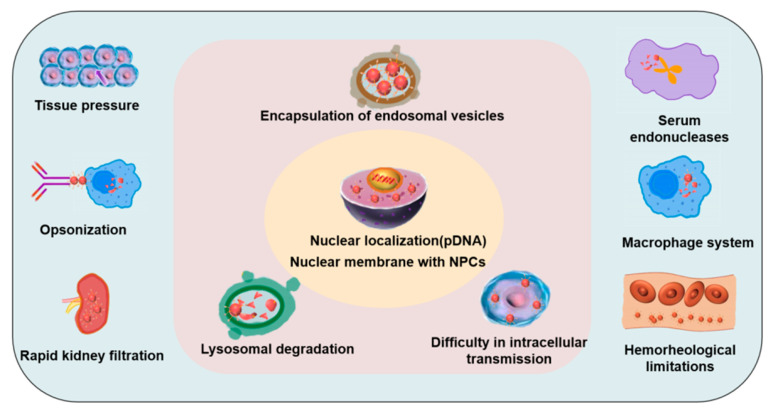
Biological barriers to CPP-based delivery systems. Biological barriers include tissue pressure, opsonization, rapid kidney filtration, serum endonucleases, a macrophage system, and hemorheological limitations. After the delivered macromolecular drugs enter the target cells, the biological barriers mainly include the encapsulation of endosomal vesicles, lysosome degradation, and the difficulty in intracellular transmission. The delivery system loaded with pDNA must enter the nucleus to function; thus, the NPC is another barrier to the pDNA delivery system.

Aside from the complex cellular uptake mechanisms, the transport of macromolecule-contained vesicles is also hindered by a variety of biological barriers in the delivery process, such as tissue pressure [93], opsonization, rapid kidney filtration, serum endonucleases, the macrophage system, hemorheological limitations [94], encapsulation of endosomal vesicles, lysosomal degradation, and intracellular transmission difficulties (Figure 5). The delivery system, loaded with pDNA, must enter the nucleus to function. As the gatekeeper of the cell nucleus, large protein assemblies, called nuclear pore complexes (NPCs), control the entry of genetic material into the nucleus [95]. NPCs are the last barrier to the pDNA delivery system. Therefore, the resolution of these obstacles needs to be further studied using optimization strategies.

## 5. Optimization Strategies of CPP-Based Delivery Systems

### 5.1. Enhancing the Endosomal Escape

Owing to the low endosomal escape efficiency of most non-viral delivery vehicles [96], encapsulation has been recognized as a key challenge for macromolecular drug delivery systems [97]. Even though one study reported that non-viral vehicles modified with CPP may inhibit drug degradation in lysosomes [98], the endosomal escape ability of most non-viral vehicles remains limited. Generally, the pH buffering effect of the proton group causes instability of the endosomal membrane or fusion with the lipid bilayer of the endosome. This increases the endosomal escape of non-viral vehicles such as membrane cleavage peptides and polymers, lysosome agents, and fusion lipids [99]. Among them, pH-sensitive peptides and polymers are widely used for endosomal escape [100,101,102,103]. In addition, Shiroh Futaki’s team developed a lipid-sensitive endosomal lytic peptide that is totally different from the pH-sensitive cleavage peptide, and it was successfully used for antibody delivery [85].

Using the CPP delivery strategy, combined with endosomal escape, Xia Ningshao’s team proposed a multifunctional chimeric peptide eTAT bio-macromolecular delivery system that greatly enhances the lysosome escape efficiency and delivery efficiency; it was successfully used in a mouse model with acute liver failure [104]. The protein delivery system consists of four modules in series: a CPP (TAT), a pH-dependent membrane-active peptide (INF7), endosome-specific protease sites, and a leucine zipper. The acidification allosteric of INF7 can destroy the endocytic vesicle membrane, and then with endosome-specific protease hydrolysis and cleavage, macromolecular protein escapes from the destroyed endosomal membrane and dimerizes through the leucine zipper, enhancing the serum tolerance, and further increasing the number of macromolecular cargos that escaped from the endosome [104]. In addition, polyethyleneimine (PEI) plays a unique proton sponge role that promotes endosome escape to a certain extent; it is usually used in combination with other components in the delivery system. In the CPP-based delivery system, PEI combined with other effectors can deliver siRNA successfully and can inhibit various kinds of tumors [105]. Since PEI still has cytotoxic and non-biodegradable properties [106,107], further optimization strategies have been proposed to overcome them [108,109].

### 5.2. Extending Half-Life in Blood

The shape and size of CPP-based vehicles can be designed to extend their half-life in the blood. It is believed that complex nanoparticles or nanovehicles at around 100 nm are an ideal choice for prolonging their half-life in the blood. Exosomes derived from living cells have excellent biocompatibility, low immunogenicity, and good delivery capabilities [110,111]. Exosomes hold great promise to serve as vehicles for targeted drug delivery. Loading superparamagnetic iron oxide nanoparticles (SPIONs) and curcumin (Cur) into exosomes and then conjugating the exosome membrane with neuropilin-1-targeted peptide (RGERPPR, RGE) by click chemistry can be used to obtain glioma-targeting exosomes with imaging and therapeutic functions [110,111]. Mechanical extrusion of approximately 10^7^ cells grafted with lipidated ligands can generate cancer cell-targeting extracellular nanovesicles (ENV). And aptamer-conjugated nanovesicles have been used for targeted drug delivery [110,111]. By using arginine-rich CPPs to induce active macropinocytosis, the cellular uptake of CPP-modified EV can be enhanced. The induction of macropinocytosis via a simple modification to the exosomal membrane using stearylated octaarginine, which is a representative CPP, significantly enhances cellular EV uptake efficacy. Consequently, effective EV-based intracellular delivery of an artificially encapsulated ribosome-inactivating protein, saporin, in EVs was attained [112]. By designing a novel peptide-equipped exosome platform that can be assembled under convenient and mild reaction conditions, the Bangce Ye team was able to bind the HepG2 cell-derived exosome surface to CPP (R9) [113]. This not only enhances the penetrating capacity of exosomes but also assists exosomes in loading ASOs. Interestingly, such a CPP-equipped delivery system remarkably increases the delivery of ASO G3139 to silence anti-apoptotic protein Bcl-2 [113].

### 5.3. Targeting CPPs

The targeting CPPs contain peptide sequences that have natural targeting abilities and can penetrate the cell membrane by interacting with the cell surface receptors when they reach a specific site [114]. Adding a targeting moiety could reduce the side effects by reducing the accumulation of drugs in non-targeted tissues; however, it may increase the complexity of peptides, the difficulty of synthesis, and the price [115]. Therefore, the target sequence should be added with caution. Several targeting CPPs are introduced below, including R6LRVG, tLyP-1, and iRGD.

#### 5.3.1. R6LRVG Targeting CPP

Oral medication is simple and convenient for patients, and the slow-release absorption of the drug reduces the side effects [116]. R6LRVG targeting CPP is an intestinal targeting penetrating peptide that consists of CPP (R6) and LRVG peptide [117]. Usually, LRVG peptide is used to target the intestinal epithelial cells in order to improve the oral bioavailability of drugs. Tyroserleutide (YSL), a tripeptide extracted from pig spleen, can suppress the invasion of mouse cancer cells [118]. Based on this evidence, Liefeng Zhang’s team designed YSL-PLGA nanoparticles modified by R6LRVG (YSLPLGA/R6LRVG nanoparticles) and enhanced the absorption of YSL for oral anti-tumor therapy [119].

#### 5.3.2. tLyP-1 Targeting CPP

Tumor-specific CendR peptides contain a tumor-homing motif and a cryptic CendR (C-terminal C-end Rule) motif that is proteolytically unmasked in tumor tissue. The cyclic tumor-homing peptide, LyP-1 (CGNKRTRGC), contains a CendR element and can penetrate tissue. In the truncated form of LyP-1, in which the CendR motif is exposed (CGNKRTR; tLyP-1), both LyP-1 and tLyP-1 internalize into cells through the neuropilin-1-dependent CendR internalization pathway. The targeted penetrating peptide tLyP-1 can promote tissue penetration through NRP-1-dependent Cend rule (CendR) internalization, thereby increasing the delivery of macromolecular drugs to the target tissue [120,121]. Guo Yuan and Qiu Zeng’s team designed a peptide-functionalized dual-targeted delivery system that encapsulates paclitaxel and GANT61 (an inhibitor of SHH signaling pathway) in a tLyP-1 peptide-modified reconstituted high-density lipoprotein nanoparticle (tLyP-1-rHDL-PTX/GANT61 NP) for metastatic triple-negative breast cancer (TNBC) treatment. The apolipoprotein A-1 and tLyP-1 peptide, modified on the surface of nanoparticles, enable the delivery system to target tumor cells by binding to the overexpressed scavenger receptor B type I and neuropilin-1 receptor. Moreover, the tLyP-1 peptide also enables the deep tumor penetration of nanoparticles, thus further facilitating paclitaxel and GANT61 delivery. In metastatic TNBC model, such a nanoparticle delivery system successfully inhibited the growth and metastasis of a primary tumor, and it has good biological safety [122].

#### 5.3.3. iRGD Targeting CPP

iRGD (CRGDKGPDC) is a cyclic tumor-targeting penetrating peptide, composed of a tumor-specific RGD motif (recognizing highly expressed integrins in cancer vasculature) and a CendR motif (which can enhance vascular permeability) [39]. Because iRGD has tumor-targeting properties and can efficiently penetrate the blood-brain barrier (BBB) [123,124], it has been extensively studied. Yunfeng Lin’s team used iRGD to enhance targeted delivery to tumor sites. Briefly, Doxorubicin (DOX), iRGD-NH2, and tetrahedral framework nucleic acid (tFNA) are mixed in sequence to prepare tFNA/DOX@iRGD, which can target αv integrin-positive breast cancer cells and transfer them to lysosomes to release cytotoxicity [125]. The iRGD modification enhances the penetration of tFNA, which is used to target the delivered chemotherapeutics to triple-negative breast cancer. iRGD can also be used to improve the radiotherapy of glioblastoma [124]. The iRGD-modified ultrasmall single-crystal Fe nanoparticles, combined with immune checkpoint blocking immunotherapy, promoted ferroptosis and immunotherapy [126]. In addition, the Ppa-iRGDC-BK01 (iRGD derivative) can exert a multiple-quenching effect on the photosensitivity. This effectively prevented non-specific phototoxicity in the light-responsive nano-delivery system [127].

### 5.4. Stimuli-Responsive Strategies

**Figure 6:**
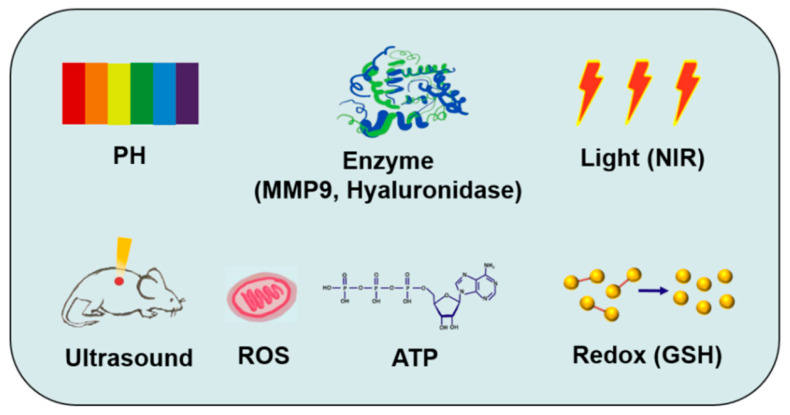
The main stimuli-responsive strategies of CPP-based macromolecular drug delivery. This figure lists the main stimulus factors used in stimuli-responsive strategies of CPP-based delivery systems in recent years, including pH, enzyme, light, ultrasound, ROS, ATP, and redox.

Stimuli-responsive strategies are designed for the characteristic differences between target organizations and non-target organizations. Different factors, including pH value, enzyme activity, light radiation, reactive oxygen species (ROS), redox potential, adenosine triphosphate, temperature gradient, ultrasonic energy, and magnetic field, are utilized to design activatable CPPs (ACPPs) [128,129]. Combining stimulus-responsive strategies can greatly improve the targeting delivery system and can enhance the therapeutic effect of diseases. Several stimuli-responsive strategies have been integrated with CPP-based macromolecular drug delivery systems (Figure 6 and Table 1).

#### 5.4.1. pH-Responsive Strategy

Generally, the acidity of tissues in pathological conditions (e.g., malignancy) is higher than that of normal tissues. The pH values of different intracellular substructures also differ. For example, endosomes and lysosomes have a higher acidity than the remaining subcellular sites [130]. Therefore, pH-sensitive nanoparticles can utilize this low-pH microenvironment to deliver therapeutic macromolecular drugs to target tissues [131]. This pH-responsive strategy has been extensively studied in the field of therapeutic delivery. Generally, pH-responsive nanoparticles can be synthesized by introducing ionizable pH-sensitive functional groups (such as amines or carboxylic acids) or acid-labile chemical bonds (such as hydrazone or ester bonds) with a specific acidity [132,133]. In a CPP-based macromolecular drug delivery system, Chunmeng Sun’s team combined CPPs (R6) with pH-responsive nanoliposomes to deliver Artemisinin to inhibit tumor growth in mice with breast cancer [134]. Four CPP-activation strategies are frequently used to design pH-responsive macromolecule delivery: (1) pH-sensitive linker; (2) pH-sensitive conformational change; (3) pH-sensitive charge conversion; and (4) pH-sensitive side-chain modification (Table 1). These strategies mainly aim to remove the inhibitory domains or change the residue modifications that sterically mask the CPP.

#### 5.4.2. Enzyme-Responsive Strategy

Enzymes play an important role in biological metabolism, with a high degree of selectivity and specificity [135]. Owing to the high expression of matrix metalloproteinase (MMP), hyaluronidase (HAase), proteolytic enzymes, and other extracellular enzymes in tumor tissues, developing macromolecular drug delivery systems that respond to different enzyme concentrations have been promoted [136].

Through integrating an MMP-responsive strategy into a CPP-based delivery system, Gao Jing’s team designed an integrated hybrid nanovesicle composed of cancer cell membranes (Cm) and matrix metallopeptidase 9 (MMP-9)-switchable peptide-based charge-reversal liposome membranes (Lipm) that coat lipoic acid-modified polypeptides (LC) co-loaded with phosphoglycerate mutase 1 (*PGAM1*) siRNA (siPGAM1) and DTX. This hybrid nanovesicle comprises a cancer cell membrane (enhancing the ability of homologous targeting) and a CPP-bound MMP-9 sensitive peptide (R9-PVGLIG-EGGEGGEGG) that carries DTX, lipoic acid-modified Polypeptide (LC) and siRNA of *PGAM1* (a key aerobic glycolytic enzyme in cancer metabolism) to treat lung cancer [137]. This delivery system exhibits a favorable negative surface charge that maintains the stability of systemic circulation. It accumulates in tumor sites overexpressing MMP-9, exposes positively charged CPPs after MMP lysis, and leads to enhanced internalization in the target cells [138]. Moreover, this delivery system has no obvious toxicity and successfully prolongs the life span of tumor-xenografted mice. Hyaluronic acid (HA) is the main endogenous ligand of CD44. It is usually used to construct hyaluronidase-responsive vehicles and to deliver siRNA [139], pDNA [140], and other genetic materials. Nanoparticles modified with HA can effectively target cancer cells that overexpress CD44 [81]. To integrate the hyaluronidase-responsive strategy into a CPP delivery system, Jianping Liu’s team developed hyaluronic acid (HA)-coated LOX-1-specific siRNA-condensed CPP nanocomplexes (NCs) and showed that HA-coated CPP NCs were promising as nanovehicles for efficient macrophage-targeted gene delivery and antiatherogenic therapy [141]. They also revealed that macrophages internalized these types of NCs via the caveolae-mediated endocytosis pathway [141]. Tingjie Yin’s team presented a biomineralization-inspired dasatinib (DAS) nano drug (CIPHD/DAS) that sequentially overcomes all the abovementioned hindrances for the efficient treatment of solid tumors. CIPHD/DAS exhibited a robust hybrid structure constructed from an iRGD-modified hyaluronic acid-deoxycholic acid organic core and a calcium phosphate mineral shell. This optimized strategy, with sequential permeabilization of the capital “leakage obstacles”, validates a promising paradigm that overcomes the “impaired delivery and penetration” bottleneck associated with nano drugs in the clinical treatment of solid tumors [142]. Aside from the MMPs and hyaluronidase, cathepsin, elastase, autophagy-specific enzymes, and aminopeptidase have also been utilized to trigger the removal of an inhibitory domain or to change the residue modification that masks the CPP activity (Table 1).

#### 5.4.3. Light-Responsive Strategy

The tumor vascular system is characterized by a poor structure, uneven branching, and uneven distribution, which makes the spatial distribution of nanovehicle-based macromolecular drugs disorderly [143]. Usually, a light-responsive strategy has a good effect on tumor treatment by directly acting on the tumor site. The stimuli used for light response strategies usually include ultraviolet light (UV; 10–400 nm), visible light (400–750 nm), and near-infrared light (NIR; 750–900 nm), among which NIR is widely used to integrate with CPP-based drug delivery systems. Yang Ding’s team proposed functionalized peptide–lipid hybrid particles, which were applied to encapsulate a PLGA polymeric core together with indocyanine green (ICG) and packaged by a lipoprotein-inspired structural shell. To initiate the precision tumor-penetrating performance, tLyP-1-fused apolipoprotein A-I-mimicking peptides (D4F) were utilized to impart tumor-homing and tumor-penetrating biological functions. The sub-100 nm drug vehicle possessed a long circulation time with uniform mono-disparity; however, it was stable enough to navigate freely, penetrate deeply into tumors, and deliver its cargo to the targeted sites. Moreover, ICG-encapsulated penetrable polymeric lipoprotein particles (PPL/ICG) could achieve real-time fluorescence/photoacoustic imaging and could monitor the in vivo dynamic distribution. Upon NIR laser irradiation, PPL/ICG exhibited a highly efficient phototherapeutic effect that eradicated the orthotopic xenografted tumors with good biosafety [144]. These external triggers, such as UV light, have also been explored to activate different types of CPPs by removing the inhibitory domain or changing the residue modification or conformational transition; however, they were validated relatively less when in vivo (Table 1).

#### 5.4.4. ROS-Responsive Strategy

Generally, ROS levels would be significantly increased due to an abnormal metabolism in a variety of pathological conditions, including diabetes, cancer, premature senescence, and neurodegenerative diseases [145]. The ROS-responsive strategy for the site-specific delivery of macromolecular drugs was developed by exploiting the differences in ROS levels between healthy and pathological tissues. Tumor cells overexpress FGL1 and PD-L1, which, respectively, bind to LAG-3 and PD-1 on T cells, forming important signaling pathways (FGL1/LAG-3 and PD-1/PD-L1) that negatively regulate immune responses. In order to interfere with the inhibitory function of the FGL1 and PD-L1 proteins, Xue-nong Zhang’s team designed a new type of ROS-sensitive nanoparticle to load *FGL1* siRNA (siFGL1) and *PD-L1* siRNA (siPD-L1), which formed a stimuli-responsive polymer with a poly-l-lysine-thioketal and modified cis-aconitate to facilitate endosomal escape. Moreover, the tumor-penetrating peptide iRGD and the ROS-responsive nanoparticles were co-administered to further enhance the delivery efficiency of si*FGL1* and si*PD-L1*, thereby significantly reducing the protein levels of FGL1 and PD-L1 in tumor cells. This nanoparticle construction had good tumor microenvironment responsiveness, and the delivery efficiency was sharply enhanced [146]. Most ROS-responsive strategies are designed by introducing a 4-boronic mandelic acid moiety between a cationic CPP and an anionic inhibitory domain (Table 1).

#### 5.4.5. Other Responsive Strategies

The integration of an ultrasound-responsive strategy into a CPP-based delivery system has also rapidly developed in recent years. Among the ultrasound-responsive strategies, low-intensity focused ultrasound (LIFU) is widely used. JianLi Ren’s team successfully developed novel tumor-homing-penetrating peptide-functionalized drug-loaded phase-transformation nanoparticles (tLyP-1-10-HCPT-PFP NPs) for LIFU-assisted tumor ultrasound molecular imaging and precise therapy. Induced by the tLyP-1 peptide with targeting and penetrating efficiency, the tLyP-1-10-HCPT-PFP NPs could increase tumor accumulation and penetrate deeply into the extravascular tumor tissue, penetrating through the extracellular matrix and the cellular membrane into the cytoplasm. With LIFU assistance, the tLyP-1-10-HCPT-PFP NPs could phase-transform into microbubbles and enhance the tumor ultrasound molecular imaging for tumor diagnosis [147]. The ATP-responsive strategy has also been integrated with a CPP-based macromolecular drug delivery system. For example, Kaiyong Cai’s team reported an adenosine triphosphate (ATP)-responsive nanovehicle with zeolitic imidazolate framework-90 (ZIF-90) for breast cancer combination therapy. Briefly, Atovaquone (AVO) and hemin are loaded into ZIF-90; then, the iRGD peptide is modified on the ZIF-90 nanoplatform. Hemin can specifically degrade BTB and CNC homology1 (BACH1), consequently changing the mitochondrial metabolism; AVO acts as the inhibitor of the electron transport chain (ETC). The degradation of BACH1 using Hemin can effectively improve the anti-tumor efficiency of the mitochondrial metabolism inhibitor, AVO, by increasing its dependency on mitochondrial respiration. This nano platform displays both tumor-targeting and mitochondria-targeting capacities, along with a high level of ATP-responsive drug release behavior and limited side effects [148]. In addition, it was reported that the strategies regarding the hypoxia-responsive fusion/conjugate constructs and the GSH-responsive release of the inhibitory domain could be used to design an ACPP-based macromolecular delivery system (Table 1).

### 5.5. Multiple Stimuli-Responsive Strategies

Monotherapy, with a continuous low dose, usually cannot completely suppress tumor growth; consequently, it is easy to increase the risk of metastasis and drug resistance [149,150]. Therefore, the combined use of multiple stimuli-responsive therapies may have a better therapeutic effect. For example, Tingjie Yin’s team combined hyaluronidase-responsive, light-responsive, and tumor-targeted peptide strategies to achieve a focus-specific penetrated delivery with photothermal therapy-mediated chemosensitization and photothermal therapy-strengthened Integrin targeting. By combining polyethylene glycol (PEG), hyaluronic acid (HA), and iRGD-modified graphene oxide (GO), they constructed iRGD-modified GO nanosheets (IPHG). The IPHG can be actively transported through the vasculature, significantly improving the infiltration of drugs in tumors. After the tumors infiltrated by IPHG-DOX are exposed to NIR stimulation, the induced photothermal effect makes the tumors susceptible to chemotherapy and inhibits cytoskeleton remodeling. Consequently, IPHG-DOX significantly inhibited the growth and metastasis of breast cancer in situ, and it could be used to prevent tumor resistance and metastasis caused by poor chemotherapy targeting [151].

The combination of redox-responsive and light-responsive nanoparticles with CPPs has also been developed to enhance chemo-photodynamic therapy. For example, by co-administering tumor-penetrating peptide iRGD and GSH-responsive SN38-dimer (d-SN38)-loaded nanoparticles, a gradual stimulation response strategy was developed [152]. These nanoparticles were transformed into nanofibers concomitantly when the tumor site was irradiated by laser to promote their retention in the tumor and the burst release of ROS for photodynamic therapy. D-SN38, loaded with disulfide bonds, responds to high levels of GSH at the tumor site, resulting in the release of SN38 and excellent chemo-photodynamic effects. This enhanced chemo-photodynamic therapy effect produced high anti-tumor effects in breast tumor models [152]. In addition, to achieve synergistic chemodynamic therapy and chemotherapy, the iRGD-modified theranostic nano drug (iRPPA@TMZ/MnO) contains pH-responsive and redox-responsive manganese oxide, which provides a new type of therapeutic, diagnostic nanomedicine for brain MRI diagnosis and the treatment of glioma [123]. There are also additional stimuli-responsive strategies based on the ultrasound response. For example, Jianping Liu’s team developed a TAT-based hyaluronidase-responsive strategy combined with ultrasound to provide a new method for the precise treatment of liver cancer [141].

All in all, CPPs can be unidirectionally inactivated by introducing inhibitory domains or when bulky groups are undesired, side-chain modifications can be used to mask the CPP activity. Masking groups can be removed by local triggers, such as enzymes or a low pH, as well as external triggers, such as light. Aside from these temporary activations, reversible activation has also been achieved by controlling the conformation of CPPs. Notably, the triggers used to activate CPPs are not so binary when in vivo. Some enzymes that are overexpressed in diseased tissue are also expressed in lower amounts in healthy tissue, and gradient pH variations can also be observed between tissues. Light-triggered CPP activation is beneficial to create temporal and special control; however, it is challenged by the poor tissue penetration depth as well as the potential cellular toxicity induced by the harmful wavelengths. Considering the heterogeneity and complexity of a disease microenvironment, multiple stimuli-responsive strategies may be more promising for designing CPP-based macromolecular drug delivery systems (Table 1).

**Table 1:** Stimuli-responsive strategies integrated with CPP-based macromolecular drug delivery.

Responding Strategy	Activatable/Specific Moiety	Loaded Drugs	CPPs	Disease Model	Refs
pH-responsive	pH-sensitive linker	Irinotecan- and *miR-200*-loaded liposomes and lipid nanoparticles	RF CPP: LKARFH. NG2 targeting the H peptide. Mitochondria targeting the K peptide. (PEG-lipid derivative with an imine bond confers pH-responsive release, internalization, and intracellular distribution in acidic microenvironment)	Colon cancer	[153]
	pH-sensitive linker	*PLK-1* siRNA-loaded liposome	ehGehGehGehG-(hydrazone)-RRRRRRRR. (Low pH triggers hydrazones to hydrolyse, resulting in loss of the inhibitory domain)	N/A	[154]
	pH-sensitive conformational change	DOX-loaded micelle	PLA-PEG -polyHis-GCGGGYGRKKRRQRRR. (Imidazole confers histidines that act as a pH trigger. Low pH protonates histidine, causing it to lose its hydrophobic interactions and the exposed Tat)	Ovarian cancer, breast cancer, and lung cancer	[155]
	pH-sensitive conformational change	PTX-loaded liposome	AGYLLGHINLHHLAHL(Aib)HHILC. (the H side-chain charges. Endowed pH responsiveness after complete replacement of all lysine in the sequence with histidine)	Colon cancer	[156]
	pH-sensitive conformational change	PET- and SPECT-probes, gold nanoparticles, and magnetic nanoparticles	pHLIP-var3: ACDDQNPWRAYLDLLFPTDTLLLDLLW. pHLIP-var7: ACEEQNPWARYLEWLFPTETLLLEL. (low pH insertion peptide pHLIP reversibly folds and is inserted across membranes in response to pH changes)	Cervix cancer, lung cancer, pancreatic cancer	[157,158]
	pH-sensitive conformational change	PTX	(LHHLCHLLHHLCHLAG)_2_. (Disulfide oxidation forms LH_2_ dimeric peptide. Lysine is substituted for histidine for endosomal escape, and the dimeric form of amphipathic CPPs shows enhanced CPP activities)	Breast cancer	[159]
	pH-sensitive charge conversion	ART-loaded liposome	HEHEHEHEHEHEHEHEHEHEGGGGGRRRRRR. (the histidine-glutamic acid-based masking peptide is modified to R6 via a spacer of 5-mer glycine)	Breast cancer	[134]
	pH-sensitive charge conversion and structure shift	TRAIL- and PTX-co-delivered liposomes	_C_(RGDfK)-AGYLLGHINLHHLAHL(Aib)HHIL-Lys-C18. (a histidine-rich peptide for pH responsiveness, c(RGDfK) peptide for αvβ3 binding, and stearyl chain C18 for membrane anchoring)	Melanoma	[160]
	pH-sensitive side-chain modification	DOX	CRRRRRRRRGGGPKKKKKK. (Conjugated DMA to lysine induces intramolecular electrostatic interactions with arginine, thereby inactivating ACPP. Low pH triggers labile amides that are hydrolyzed)	Liver cancer	[161]
	pH-sensitive side-chain modification	DOX-loaded PEG-PCL micelle	YGR^a^K^a^KRRQRRRC. (Amidized CPPs. Conjugated succinyl moieties to the glutamine and both lysine residues of Tat)	Ovarian cancer	[162]
Enzyme-responsive	MMP-9-sensitive linker	DNase I- and PTX prodrug-loaded NET-regulated nanoparticle	GRKKRRQRRRPQPLGLAGGC. (MMP-9 substrate peptide linked to Tat)	Breast cancer, lung cancer	[163]
	MMP9-sensitive linker	CsA-loaded, MMP-9-sensitive CPP-decorated reconstituted lipoprotein nanoparticles	ACFAEKFKEAVKDYFAKFWDGSGRRRRRRRRRPVGLIGEGGEGGEGG. (MMP-9 substrate peptide conjugating with APOA-I mimics α-helix peptide through a GSG linker)	Traumatic brain injury	[138]
	MMP9-sensitive linker	*PGAM1*-siRNA- and DTX-loaded nanovesicles	RRRRRRRRRPVGLIGEGGEGGEGG.	Lung cancer	[137]
	MMP-2 & -9-sensitive linker	Cy5, Gadolinium chelates	EEEEEEEE-PLGLAG-RRRRRRRRR. EEEEEE-PLGLAG-RRRRRRRRR. (polycationic CPP is coupled via a cleavable linker to a neutralizing peptide)	Image-guided surgery of different kinds of tumors	[164,165]
	HAase-sensitive linker	HA-coated, *LOX-1*-siRNA-loaded nanocomplexes	RQIKIWFQNRRMKWKK.	Atherosclerosis	[141]
	Cathepsin-sensitive linker	Dox-loaded SiO2 nanoparticles	EEEEEEPGFKYGRKKRRQRRR.	Lung cancer, ovarian cancer	[166]
	Elastase-sensitive linker	Cy5	EEEEEEEEE-RLQLK(Ac)L-RRRRRRRRR.	Breast cancer	[167]
	PSA-sensitive linker	*PLK-1* siRNA-loaded liposomes	DGGDGGDGGDGG-HSSKYQ-RRRRRRRR. (PSA is serine protease)	Prostate cancer	[168]
	ATG4B-sensitive linker	DTX and CQ–loaded nanoparticles	GTFGFRRRRRRRRR. (Autophagy-specific enzyme ATG4B substrate linked to R9)	Melanoma	[169]
	APN-DPP4-sensitive side-chain modification	FITC	GRKKRRQRRRAhxC (Side chain modifications. Aminopeptidase N dipeptidyl peptidase IV)	N/A	[170]
Hypoxia-responsive	Oxygen-sensitive degradation of fusion protein	ODD-beta-Gal	YGRKKRRQRRR-ODD-Casp3(wt) (Tat-oxygen-dependent degradation domain-Caspase 3 fusion protein is selectively stabilized in hypoxic tumors)	Pancreatic cancer	[171]
	Azoreductase-sensitive modification	Peptide nucleic acid (PNA)	MVTVLFRRLRIRRACGPPRVRV-azo-PEG (Activatable CPP-PEG conjugates. Azoreductase-triggered CPP-inactivation through functionalization with a self-immolative azobenzene moiety)	Colon mucosa	[172]
ROS-responsive	ROS-sensitive polymer	*FGL1*-siRNA, PD-L1-siRNA	c(CRGDKGPDC) (Proteolysis of iRGD peptide exposes a new motif that can bind to NRP-1 and activate neuropilin, allowing drugs or nanoparticles to leak out from tumor blood vessels and penetrate the tumor tissue.)	Liver cancer	[146]
	ROS-sensitive linker	FITC, Cy5	EEEEEEEEE-cleavable linker-RRRRRRRRR. (H_2_O_2_-activated CPP. A boronic acid-containing cleavable linker between polycationic CPP and polyanionic fragments)	Lung inflammation	[173]
ATP-responsive	ATP-sensitive release of guest molecules	Photosensitizers	Ac-QYFMpTEpYVA (ATP-triggered release of phosphopeptides from the pegylated GC5A-12C nanocarrier (12C-NC) system. Host-guest ATP-responsive system)	N/A	[174]
	ATP-sensitive disintegration	Atovaquone (AVO), hemin	c(CRGDKGPDC)-ZIF-90 (iRGD peptide-modified ZI-90/protein nanoparticles disintegrate in the presence of ATP to release protein as a result of the competitive coordination between Zn2+ and ATP)	Breast cancer	[148]
Ultrasound-responsive	Ultrasound-assisted phase-Transformation	Hydroxycamptothecin	CGNKRTR. (Tumor homing-penetrating peptide-functionalized drug-loaded phase-transformation nanoparticles tLyP-1-10-HCPT-PFP)	Breast cancer	[147]
	Ultrasound-activated cavitation effect	Pefluoropentane, 10-Hydroxycamptothecin-loaded liposome nanoparticle	CGNKRTR. (Truncated form of LyP-1 CPP (CGNKRTRGC))	Breast cancer	[175]
	Ultrasound-dependent endosomal escape	shRNA	Tat-U1A-rose bengal conjugate (Tat cell-penetrating peptide, U1A RNA-binding protein, and rose bengal as a sonosensitizer)	N/A	[176]
GSH-responsive	GSH-sensitive disulfide linker	Podophyllotoxin (PPT), Doxorubicin	PRASHANT. (anti-mitotic PRA octapeptide-linked PPT conjugate that can self-assemble into a vesicle via water and targeted synergistic drug delivery)	N/A	[177]
Light-responsive	UV light-sensitive self-immolative linker	Doxorubicin	ARTKQTARKSTGGKAPRKQLATKAARKSAPATGGC^35^KKPHRYRPGTVALREIRRYQKSTELLIRKLPFQRLVREIAQDFKTDLRFQSSAVMALQEASEAYLVALFEDTNLAAIHAKRVTIMPKDIQLARRIRGERA. (H3-^35^PC4AP. PC4AP (a photo-caged C4’-oxidized abasic site) as a light-responsive, self-immolative linker to conjugate drugs to a CPP)	N/A	[178]
	UV light-sensitive linker	Quantum dots, polystyrene particles, Au nanostars, and liposomes	RRRRRRR-o-nitrobenzyl-GGGEEEEEEE. (a photo-caged peptide that undergoes a structural transition from an antifouling ligand to CPP upon photo-irradiation)	N/A	[179]
	NIR-sensitive side-chain modification	VB-loaded liposome	CGRRMK^PG^WK^PG^K^PG^. NGR peptide: CYGGRGNG; Synergistic effect (light-released photolabile-protective group PG (4,5-dimethoxy-2-nitrobenzene chloroformate))	Fibrosarcoma	[180]
	UV-sensitive side-chain modification	Proapoptotic peptide (KLAKLAK)_2_	Ac-KRRMK^Nvov^WK^Nvoc^K^nvoc^. (Nvoc=6-nitroveratrylcarbonyl; light-activated caged Pen CPP; photo-cleavable groups)	N/A	[181]
	UV-sensitive conformational change	Tamra	cis-Ab-LK. Trans-Ab-LK azobenzene (Ab) linker	N/A	[182]
	UV/Vis-sensitive conformational change	RhoB	RRRRRRRRR-AB-EEEEEEEEE. (cis-to-trans isomerization of azobenzene (AB) moiety; photoswitchable)	N/A	[183]
	UV light-sensitive linker inhibitory domain	Atto655-loaded liposome	YGAKKARQRRAGC-PEG-loop. (modified on both termini of Tat with an alkyl chain; UV-cleavable linker)	N/A	[184]
Multiple-responsive	NIR- and pH-dual sensitive linker	*EGFR* siRNA	CGRRMKWKK-DMNB-EEEERRRR. (CPP is quenched by a pH-sensitive inhibitory peptide, which is linked via a photo-cleavable group DMNB)	Breast cancer	[185]

## 6. Clinical Challenges of CPP-Based Macromolecular Drug Delivery

Several types of clinical trials have involved CPP-based macromolecular drug delivery (Table 2). For example, as a c-Jun N-terminal Kinase (JNK) inhibitor, the AM-111 penetrating peptide (brimapitide) drug is being tested to treat acute unilateral sudden deafness. The XG-102 drug (brimapitide), a TAT-coupled dextrogyre peptide that selectively inhibits the c-Jun N-terminal kinase for treating postoperative ocular inflammation, has entered Phase III clinical trials [186,187]. In addition, TAT-based class A botulinum toxin drugs have also been extensively explored in clinical practice [188,189]. Although several trials of initial CPP-based macromolecular drugs have been discontinued, advanced CPP-integrated macromolecules are still actively tested in clinics (Table 2).

**Figure 7:**
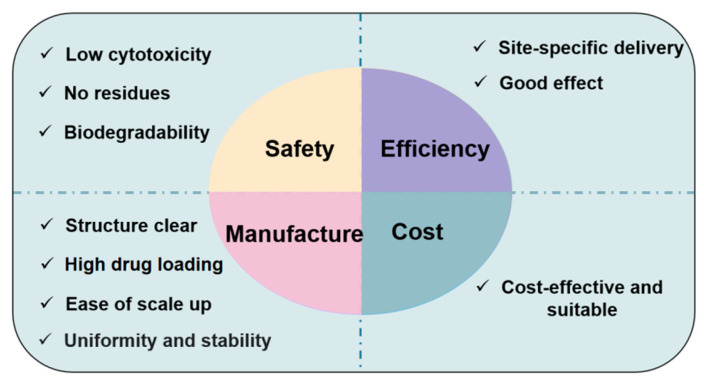
Conditions for successful CPP-based macromolecular drug delivery. In the experimental phase of the delivery system, safety and efficiency are the main concerns. In the final application to the clinic, manufacturing and cost will be essential factors to be considered. Successful CPP-based delivery of macromolecular drugs must concomitantly satisfy the conditions of safety, efficiency, manufacturing, and cost.

However, to date, the macromolecular drugs formulated with a CPP-based delivery system have not been formally approved by the FDA for marketing. Therefore, we proposed that a successful CPP-based macromolecular drug delivery system must be safe (low cytotoxicity, no residues after its action, and biodegradable), effective (a strong specificity and good effects), manufacturable (high drug-loaded, with a clear structure for scale-up, and uniform and stable materials), and cost suitable (Figure 7).

## 7. Challenges and Future Directions

By comparing different CPP-based macromolecular drug delivery platforms, this review deconstructs the literature into a comprehensive and understandable framework. (1) The source and classification of CPPs are introduced in detail; then, the cellular uptake mechanisms and influencing factors are analyzed based on the CPP-based delivery system. (2) The optimization strategies of CPP-based delivery systems, including improving endosomal escape, prolonging the half-life in blood, targeting CPPs, as well as single and multiple stimuli-responsive strategies, are examined. (3) The CPP-based delivery systems have achieved good curative effects in refractory diseases, including glioma, solid tumors, and triple-negative breast cancer. After reviewing the clinical attempts, the conditions for a successful CPP-based macromolecular drug delivery system are summarized to provide reference significance.

Discoveries from a CPP-based delivery system, combined with therapeutic macromolecular drugs, have prompted renewed attention in this field. However, a successful CPP-based delivery system has not yet been developed. In recent years, research on macromolecular drug therapy has mainly focused on breakthroughs in solving the stability of and targeting CPP-based delivery systems. Specifically, targeting without off-target toxicity is the most important key to ensuring the safety and efficacy of CPP-based macromolecular drug delivery, whereas good stability is an unavoidable key that should be scaled up. Although the current review proposes the latest optimization strategies, the complex structure of CPP-based macromolecular drug delivery systems, when combined with some targeting moiety, may bring about new safety and production issues. Thus, the CPP-based drug delivery platforms remain immature and need to be further improved in the future.

## Figures and Tables

**Table 2:** CPP-based therapeutics in clinical trials.

CPPs	Cargos	Recruitment Status	Application	Gov ID	Year	Refs
TAT	Botulinum toxin A	Phase IIIb (completed)	Cervical dystonia	NCT01753310	2012	[190]
TAT	JNKI-1	Phase III (completed)	Postoperative ocular inflammation	NCT02508337, 02235272	2015 2017	[187]
TAT	PSD-95 protein inhibitor	Phase III (completed)	Ischemic stroke	NCT02930018	2016	[191]
TAT	D-JNKI-1 gel	Phase III (completed)	Hearing loss, idiopathic sudden sensorineural	NCT02809118, 02561091	2016 2015	[186]
TAT	Botulinum toxin A	Phase II (completed)	Cervical dystonia	NCT02706795	2016	[188]
TAT	δ-PKC inhibitor	Phase II (completed)	Myocardial infarction	NCT00785954	2008	N/A
TAT	ε-PKC inhibitor	Phase II (completed)	Pain: postherpetic neuralgia, spinal cord injury, postoperative	NCT01106716, 01135108, 01015235	2010 2011 2013	[192,193]
TAT	PKC inhibitor	Phase II (completed)	Acute myocardial infarction	NCT00093197	2004	[194]
TAT	Botulinum toxin A	Phase I/II	Glabellar lines	N/A	2015	[189]
TAT	Dextrogyre peptide	Phase I (completed)	Intraocular inflammation and pain	NCT01570205	2012	[195]
TAT	MAGE-A3,HPV-16	Phase I (completed)	Head and neck carcinoma	NCT00257738	2005	[196]
TAT	Cu, Zn-Superoxide dismutase	Phase I	Obesity	N/A	2011	[197]
ATX-101	N/A	Phase Ib/Iia (recruiting)	Several cancers	NCT04814875	2021	[198]
AM-111	D-JNKI-1 gel	Phase II (completed)	Acute sensorineural hearing loss	NCT00802425	2008	[199]
P28	P28GST	Phase II (completed)	Intestinal inflammation	NCT02281916	2014	[200]
P28	P28, Non-HDM2-mediated peptide inhibitor of p53	Phase I	Central nervous system tumors	NCT01975116	2016	[201]
P28	P28, Non-HDM2-mediated peptide inhibitor of p53	Phase I (completed)	P53 ubiquitination in patients with advanced solid tumors	NCT00914914	2013	[202]
ALRN-6924	Palbociclib	Phase Iia (completed)	Solid tumor, Lymphoma, Peripheral T-cell lymphoma	NCT02264613	2014	[203]
ALRN-6924	Cytarabine	Phase I (completed)	Acute myeloid leukemia, Myelodysplastic syndromes	NCT02909972	2016	
ALRN-6924	Paclitaxel	Phase 1 (active)	Advanced, metastatic or unresectable solid tumors	NCT03725436	2018	
ALRN-6924	Cytarabine	Phase 1 (active)	leukemia, brain tumor, pediatric lymphoma	NCT03654716	2018	
ALRN-6924	Topotecan	Phase 1a (terminated)	Small cell lung cancer	NCT04022876	2019	
R7	Cyclosporin A	Phase II	Psoriasis	N/A	2003	[204]
(R-Ahx-R)_4_	PMO	Phase III (terminated)	Cardiovascular disease, coronary artery bypass	NCT00451256	2007	[205]
(R-Ahx-R)_4_	PMO targeted to human c-Myc	Phase II	Obstruction of vein graft after cardiovascular bypass surgery	N/A	2009	[206]
TransMTS	Botulinumtoxin A	Phase III (completed)	Cervical dystonia	NCT03608397	2018	[188]
MTS	Botulinumtoxin A	Phase III, Phase II, Phase II (completed)	Skin aging, hyperhidrosis, lateral canthal lines, crow’s feet, and facial wrinkles	NCT02580370, 02565732	2015	[207,208]
AVB-620 (ACPP)	Cy5, Cy7	Phase II (completed)	Breast cancer	NCT03113825	2017	[209]
Pepducin	PZ-128	Phase II	Coronary artery disease	N/A	2015	[210]
AVB-620 (ACPP)	Cy5, Cy7	Phase I (completed)	Interpretative tumor detection using a ratiometric activatable fluorescent peptide	NCT02391194	2015	[211]
BT1718	Toxic DM1	Phase I/Iia (active)	Targeting MT1-MMP for treatment of solid tumors	NCT03486730	2018	[212]
PEP-010	Paclitaxel	Phase 1 (recruiting)	Metastatic solid tumor	NCT04733027	2021	
ATP128	BI 754091	Phase 1b (recruiting)	Stage IV colorectal cancer	NCT04046445	2019 2022	
PTD4	HSP20 phosphopeptide	Phase II (recruiting)	Scar prevention, reduction	NCT00825916	2009	[213]
Charged Oligo peptide	SN38	Phase I	Tumor	N/A	2016	[214]

Refs: References. Gov ID: Gov Identifier from Clinical trials.gov.

## Data Availability

Not applicable.
